# Dynamics and stationary distribution of a stochastic SIRS epidemic model with a general incidence and immunity

**DOI:** 10.1186/s13661-022-01668-0

**Published:** 2022-11-11

**Authors:** Tao Chen, Zhiming Li

**Affiliations:** College of Mathematics and System Science, Xinjiang University, Urumqi, P.R. China

**Keywords:** 37N25, 37H10, 34F05, SIRS epidemic model, General incidence, Stationary distribution, Partial immunity

## Abstract

Infected individuals often obtain or lose immunity after recovery in medical studies. To solve the problem, this paper proposes a stochastic SIRS epidemic model with a general incidence rate and partial immunity. Through an appropriate Lyapunov function, we obtain the existence and uniqueness of a unique globally positive solution. The disease will be extinct under the threshold criterion. We analyze the asymptotic behavior around the disease-free equilibrium of a deterministic SIRS model. By using the Khasminskii method, we prove the existence of a unique stationary distribution. Further, solutions of the stochastic model fluctuate around endemic equilibrium under certain conditions. Some numerical examples illustrate the theoretical results.

## Introduction

Infectious diseases have been a severe threat to human health. Many disasters in history have been due to the outbreak of contagious diseases, such as the plague pandemic, smallpox, the Black Death, AIDS, SARS [1], and recently COVID-19 [2, 3]. Mathematical models are effective ways to investigate the spread of epidemics. Kermark and McKendrick [4] first studied the dynamic behaviors of epidemics by ordinary differential equations. Since then, researchers have proposed and studied many deterministic epidemic models, see [5–12]. Suppose the total population $N(t)$ is divided into three classes at time *t*: susceptible (*S*), infective (*I*), and recovered (*R*) individuals, respectively. Based on the structure of compartments, researchers have proposed and studied some infectious-disease models according to the transmission characteristics and pathogenicity of the disease itself, such as SIR, SIRS, SIRI, and SIRIS models. For the SIR model, the recovered individuals have permanent immunity. However, for some diseases, the recovered individuals may lose immunity after a certain period and become susceptible individuals or relapse with reactivation of latent infection and revert to the infective class [13, 14]. The former can be described by the SIRS model, while the SIRI model can describe the latter. In recent years, much work has been carried out to study the SIRS model from all aspects, see [15–20]. Let $S(t)$, $I(t)$, and $R(t)$ be the number of susceptible, infectious, and recovered individuals at time *t*, respectively. Through ordinary differential equations, the deterministic SIRS model is usually expressed by

1 $$ \textstyle\begin{cases} S'(t) =\Lambda -\rho S(t)-\beta S(t)I(t)+\theta R(t), \\ I'(t) =\beta S(t)I(t)-(\rho +\eta +\alpha )I(t), \\ R'(t) =\eta I(t)-(\rho +\theta )R(t). \end{cases} $$

In the model (1), Λ denotes the recruitment rate of susceptible individuals, *ρ* and *α* are the natural and disease-related mortality rates, *β* is the contact transmission coefficient, *η* is the recovery rate, and *θ* is the immunity loss rate of recovered individuals.

In epidemic models, the incidence rate plays a vital role. It not only describes the characteristics of the disease but also measures the speed at which the disease spreads. There are two widely used incidence rates: the bilinear incidence rate $\beta SI$ [21–24] and the standard incidence rate $\beta SI/N$ [25–27]. The bilinear incidence $\beta SI$ is commonly used to model communicable diseases, for example, influenza [8, 28]. The standard incidence $\beta SI/N$ is more suitable for disease modeling when the total population is huge [1]. However, it is invalid to assume homogeneous mixing in a heterogeneous population. In this case, the transmission characteristics of the disease can be described through a suitable nonlinear incidence rate [8, 17, 29–31]. Capasso and Serio [29] introduced a saturated nonlinear-incidence rate $S\varphi (I)$ into the epidemic model. The transmission rate between the infected and susceptible will be saturated if the number of infected individuals is larger in the population. Other nonlinear incidences are used one after another, including $\beta I^{p}S^{q}$, $\beta SI^{p}/(1+\alpha I^{q})$, and $\beta I^{p}S/(1+\alpha S)$. Lahrouz et al. [8] proposed an SIRS model with a general incidence rate $\beta SI/\varphi (I)$, where *φ* is a positive function such that $\varphi (0)=1$ and $\varphi '\geq 0$. Most of the above incidence rates are the special cases of $\beta SI/\varphi (I)$. For instance, if $\varphi (I)=1$, then it is a bilinear incidence rate; if $\varphi (I)=1+\alpha I^{q}$, and the incidence rate is $\beta SI/(1+\alpha I^{q})$. Another advantage of the general incidence rate can be used to describe the psychological effect: the infection force may decrease with the number of infective individuals.

In the real world, there are kinds of infectious disease such as bacterial meningitis. Some infective individuals obtain immunity after recovery and become recovered individuals, but others have no immunity after recovery and become susceptible. This kind of character is called partial immunity. In the above kinds of SIRS models, there are two main limitations: (i) the classical SIRS model (1) with bilinear or standard incidence rates does not always effectively analyze the dynamic properties of the disease in a heterogeneous population; and (ii) although the SIRS model with a general incidence rate, proposed by [8], is more practical than the classical model, the partial immunity of infectious individuals has not been considered in the model. Generally, the individuals can be divided into two sections: one section of them after recovery have immunity and go to the recovered class. Another section after recovery has no immunity and returns to the susceptible class. For this reason, in the work we propose an SIRS epidemic model with a general incidence rate and partial immunity as follows

2 $$ \textstyle\begin{cases} S'(t) =\Lambda -\rho S(t)-\frac{\beta S(t)I(t)}{\varphi (I(t))}+(1-p) \eta I(t)+\theta R(t), \\ I'(t) =\frac{\beta S(t)I(t)}{\varphi (I(t))}-(\rho +\eta +\alpha )I(t), \\ R'(t) =p\eta I(t)-(\rho +\theta )R(t), \end{cases} $$

where *p* is the immunity rate. In the model (2), $(1-p)\eta I$ corresponds to the infected individuals who lost immunity after recovery, while $p\eta I$ represents the infected individuals who gain immunity after recovery. In particular, if $p=1$ and $\varphi (I)=1$, then the model (1) is a special case of the model (2). Define the basic reproduction number as

$$ {\mathscr{R}}=\frac{\Lambda \beta}{\rho (\rho +\eta +\alpha )}.$$

Suppose $\varphi (0)=1$ and $\varphi '(t)>0$ for $t>0$ in the model (2). Similar to [8] and [32], it is easy to obtain the dynamic properties of equilibria in the model (2) by constructing the Lyapunov function as follows:

(i) The model (2) has a unique disease-free equilibrium denoted by $E^{0}(\frac{\Lambda}{\rho},0,0)$. If ${\mathscr{R}}< 1$, the disease-free equilibrium $E^{0}$ is globally asymptotically stable.
(ii) If ${\mathscr{R}}>1$, the model (2) has a unique endemic equilibrium denoted by $E^{*}(S^{*},I^{*},R^{*})$, satisfying
  $$ \textstyle\begin{cases} \Lambda -\rho S^{*}-\frac{\beta S^{*}I^{*}}{\varphi (I^{*})}+(1-p) \eta I^{*}+\theta R^{*}=0, \\ \frac{\beta S^{*}I^{*}}{\varphi (I^{*})}-(\rho +\eta +\alpha )I^{*}=0, \\ p\eta I^{*}-(\rho +\theta )R^{*}=0. \end{cases} $$

Further, the endemic equilibrium $E^{*}$ is globally asymptotically stable. Detailed proofs of these are provided in the Appendix.

For the deterministic SIRS model (2), an important assumption is that the disease is not affected by stochastic perturbations. In practice, some parameters of the SIRS model (2) always fluctuate due to stochastic perturbations in the environment. Thus, the stochastic epidemic model can provide more realism than the corresponding deterministic models. In the past few years, many researchers have considered stochastic epidemic models and have obtained significant results [27, 30, 32–42]. For example, Jiang et al. [42] proved a global positive solution of a stochastic SIR model and investigated the asymptotic behaviors. Lahrouz and Omari [30] studied a stochastic SIRS model with a nonlinear incidence rate in a population of varying sizes. However, they did not investigate the asymptotic behavior of the solution. Zhang et al. [27] analyzed an SIRS model with a standard incidence rate under stochastic perturbations. Recently, Fatini et al. [33] analyzed a stochastic model with a nonlinear incidence and obtained the asymptotic behavior of the disease. Koufi et al. [34] considered a stochastic SIRS system with switching among different environments. Ding and Zhang [35] proposed a stochastic SIRS epidemic model with bilinear incidence. However, to the best of our knowledge, there are few reports of research about the stochastic SIRS model with general incidence and partial immunity. As an extension of the above results, we introduce various stochastic perturbations into the system (2). Then, we obtain a stochastic SIRS epidemic model with a general incidence rate and partial immunity as follows

3 $$ \textstyle\begin{cases} \mathrm{d}S = [\Lambda -\rho S-\frac{\beta SI}{\varphi (I)}+(1-p) \eta I+\theta R ]\,\mathrm{d}t+\sigma _{1}S\,\mathrm{d}B_{1}(t)-\sigma _{4} \frac{SI}{\varphi (I)}\,\mathrm{d}B_{4}(t), \\ \mathrm{d}I = [\frac{\beta SI}{\varphi (I)}-(\rho +\eta +\alpha )I ]\,\mathrm{d}t+\sigma _{2}I\,\mathrm{d}B_{2}(t)+\sigma _{4} \frac{SI}{\varphi (I)}\,\mathrm{d}B_{4}(t), \\ \mathrm{d}R =[p\eta I-(\rho +\theta )R]\,\mathrm{d}t+\sigma _{3}R\,\mathrm{d}B_{3}(t), \end{cases} $$

where $B_{i}(t)$ ($i=1,2,3,4$) are independent standard Brownian motions defined on the complete probability space $(\Omega ,{\mathscr{F}},\{{\mathscr{F}}\}_{t\geq 0},{\mathbb{P}})$ with a filtration $\{{\mathscr{F}}\}_{t\geq 0}$ satisfying the usual conditions, and $\sigma _{i}$ ($i=1,2,3,4$) are the nonnegative intensities of the standard Gaussian white noises.

The rest of the paper is organized as follows. We first review some basic concepts and useful lemmas in Sect. 2. The existence and uniqueness of the globally positive solution are proved in Sect. 3. In Sect. 4, we obtain sufficient conditions for the extinction of the disease under a stochastic system. Asymptotic behaviors of the solution are discussed around the disease-free equilibrium of the deterministic model in Sect. 5. In Sect. 6, we prove that the model (3) has a unique stationary distribution under certain conditions and discuss the asymptotic behaviors of the solution around the endemic equilibrium. A brief conclusion is provided in Sect. 7.

## Preliminaries

Let $Z(t)$ be a three-dimensional time-homogeneous Markov process described by the following stochastic differential equation (SDE)

$$ \mathrm{d}Z(t)=b\bigl(t,Z(t)\bigr)\,\mathrm{d}t +\sigma \bigl(t,Z(t)\bigr)\,\mathrm{d}B(t), $$

where $b:[t_{0},+\infty ]\times {\mathbb{R}}^{3}\rightarrow {\mathbb{R}}^{3}$, $\sigma : [t_{0},+\infty ]\times {\mathbb{R}}^{3}\rightarrow { \mathbb{R}}^{3\times 4}$ are locally Lipschitz functions in ${\mathbb{R}}^{3}$ and $B(t)=(B_{1}(t),B_{2}(t),B_{3}(t),B_{4}(t))^{\top}$ is a four-dimensional standard Brownian motion. Denote $\mathbb{R}_{+}^{3}:=\{(z_{1},z_{2},z_{3})|z_{i}>0, i=1,2,3\}$. The operator ${\mathcal {L}}$ of $Z(t)$ is defined as

$$ {\mathcal {L}}=\frac{\partial}{\partial t}+\sum_{i=1}^{3}b_{i}(t,z) \frac{\partial}{\partial z_{i}}+\frac{1}{2}\sum_{i=1}^{3} \bigl[ \sigma ^{T}(t,z)\sigma (t,z) \bigr]_{ij} \frac{\partial ^{2}}{\partial z_{i}\partial z_{j}}. $$

Denote $C^{2,1}([t_{0},+\infty ]\times {\mathbb{R}}^{3};{\mathbb{R}}_{+})$ as the family of all nonnegative functions $F(t,z)$ defined on $[t_{0},+\infty ]\times {\mathbb{R}}^{3}$ such that they are continuously once in *t* and twice in *z*. The following formula can be obtained by acting ${\mathcal {L}}$ on a function $F(t,z)\in C^{2,1}([t_{0},+\infty ]\times {\mathbb{R}}^{3};{\mathbb{R}}_{+})$

$$ {\mathcal {L}}F(t,z)=F_{t}(t,z)+F_{z}(t,z)b(t,z)+ \frac{1}{2} \operatorname{trace}\bigl[\sigma ^{\top}(t,z)F_{zz}(t,z) \sigma (t,z)\bigr], $$

where

$$ F_{t}(t,z)=\frac{\partial F}{\partial t}, \qquad F_{z}(t,z)= \biggl( \frac{\partial F}{\partial z_{1}},\frac{\partial F}{\partial z_{2}}, \frac{\partial F}{\partial z_{3}} \biggr), \qquad F_{zz}(t,z)= \biggl( \frac{\partial ^{2}F}{\partial z_{i}\partial z_{j}} \biggr)_{3\times 3}.$$

By Itô’s formula, we have $\mathrm{d}F(t,z)={\mathcal {L}}F(t,z)\,\mathrm{d}t+F_{z}(t,z)\sigma (t,z)\, \mathrm{d}B(t)$.

### Lemma 1

([43])

*Let* $\langle Q(t),Q(t)\rangle $ *be the quadratic variation of a continuous local martingale* $\{Q(t): t\geq 0\}$ *with initial value* $Q(0)=0$. *Then*, *for almost all* $\omega \in \Omega $, *there exists a random integer* $k_{0}=k_{0}(\omega )$ *such that*

$$ Q(t)\leq \frac{1}{2}m_{k}\bigl\langle Q(t),Q(t)\bigr\rangle + \frac{v\ln k}{m_{k}},\quad 0\leq t\leq n_{k}$$

*for all* $k>k_{0}$, *where* $v>1$ *is a number and* $m_{k}>0$, $n_{k}>0$ *are two sequences*.

### Lemma 2

([44])

*There exists a bounded open domain* $U\subset {\mathbb{R}}^{d}$ *with regular boundary* Γ, *having the following properties*:

1. *In the domain* *U* *and some neighborhood thereof*, *the smallest eigenvalue of the diffusion matrix* $A(z)=(a_{ij}(z))$ *is bounded away from zero*.
2. *If* $z\in {\mathbb{R}}^{d}\setminus U$, *the mean time* *τ* *at which a path issuing from* *z* *reaches the set* *U* *is finite*, *and* $\sup_{z\in K}{\mathbb{E}}\tau <\infty $ *for every compact subset* $K\subset {\mathbb{R}}^{d}$.

*If* (A.1) *and* (A.2) *hold*, *then the Markov process* $Z(t)$ *has a stationary distribution* $\pi (\cdot )$. *Further*,

$$ {\mathbb{P}} \biggl\{ \frac{1}{T} \int _{0}^{T}f\bigl(Z(t)\bigr)\,\mathrm{d}t \xrightarrow[T\rightarrow \infty ]{} \int _{{\mathbb{R}}^{d}}f(y)\pi (\mathrm{d}y) \biggr\} =1 $$

*for all* $z\in {\mathbb{R}}^{d}$, *where* $f(z)$ *be a function integrable concerning measure* *π*.

In Lemma 2, Assumption (A.1) can be verified by the existence of a positive number *M* such that $\sum_{i,j=1}^{d}a_{ij}(z)\zeta _{i}\zeta _{j}\geq M|\zeta |^{2}$, $z \in U$, $\zeta \in {\mathbb{R}}^{d}$. To verify Assumption (A.2), it is sufficient to prove that there is a nonnegative $C^{2}$-function *ψ* such that for some $\theta >0$, ${\mathcal {L}}\psi (z)<-\theta $, $z\in {\mathbb{R}}^{d}\setminus U$ (see [45]).

## Existence and uniqueness of a positive solution

In this section, we first prove that the solution of the stochastic model (3) satisfies the following properties.

### Theorem 1

*For any given initial value* $(S(0),I(0),R(0))\in \mathbb{R}^{3}_{+}$ *in the model* (3), *there exists a unique positive solution* $(S(t),I(t),R(t))$ *for* $t\geq 0$. *The solution remains in* $\mathbb{R}^{3}_{+}$ *with probability one*, *that is to say*, $(S(t),I(t),R(t))\in \mathbb{R}^{3}_{+}$ *for all* $t\geq 0$ *almost certainly*.

### Proof

Since the coefficients of model (3) are locally Lipschitz continuous, there exists a unique local solution $(S(t), I(t), R(t))$ on $t\in [0,\tau _{e}]$ for any given initial value $(S(0), I(0), R(0)) \in \mathbb{R}^{3}_{+}$, where $\tau _{e}$ is the explosion time. The solution is global if we can prove that $\tau _{e}=+\infty $ a.s. To do this, define the stopping time as

$$ \tau _{+}=\inf \bigl\{ t\in [0,\tau _{e}]: S(t)\leq 0 \text{ or } I(t)\leq 0 \text{ or } R(t)\leq 0\bigr\} . $$

Set $\inf \emptyset =+\infty $ as usual, where ∅ represents the empty set. Obviously, $\tau _{+}\leq \tau _{e}$. We now only need to prove $\tau _{+}=\infty $ a.s. If $\tau _{+}<\infty $, there must exist a positive constant *C* satisfying ${\mathbb{P}}(\tau _{+}< C)>0$. Define a function $V:{\mathbb{R}}_{+}^{3}\rightarrow {\mathbb{R}}_{+}$ as

$$ V\bigl(S(t),I(t),R(t)\bigr)=\ln \bigl(S(t)I(t)R(t)\bigr). $$

By Itô’s formula, we have

$$ \begin{aligned} \mathrm{d}V\bigl(S(t),I(t),R(t)\bigr)&= {\mathcal {L}}V \bigl(S(t),I(t),R(t)\bigr)\,\mathrm{d}t+ \sigma _{1}\,\mathrm{d}B_{1}(t)+\sigma _{2}\,\mathrm{d}B_{2}(t) \\ &\quad{}+\sigma _{3}\,\mathrm{d}B_{3}(t)+\sigma _{4} \frac{S(t)-I(t)}{\varphi (I(t))}\,\mathrm{d}B_{4}(t), \end{aligned} $$

where

4 $$\begin{aligned} {\mathcal {L}}V\bigl(S(t),I(t),R(t)\bigr)&= \frac{1}{S(t)} \biggl[ \Lambda -\rho S(t)- \frac{\beta S(t)I(t)}{\varphi (I(t))}+(1-p)\eta I(t)+\theta R(t) \biggr] \\ &\quad{}+\frac{1}{I(t)} \biggl[\frac{\beta S(t)I(t)}{\varphi (I(t))}-(\rho + \eta +\alpha )I(t) \biggr]+\frac{1}{R(t)} \bigl[p\eta I(t) \\ &\quad{}-(\rho +\theta )R(t) \bigr]-\frac{1}{2} \biggl[\sigma _{1}^{2}+ \sigma _{2}^{2}+ \sigma _{3}^{2}+\sigma _{4}^{2} \biggl( \frac{I(t)}{\varphi (I(t))} \biggr)^{2} \\ &\quad{}+\sigma _{4}^{2} \biggl(\frac{S(t)}{\varphi (I(t))} \biggr)^{2} \biggr] \\ &\geq -\rho -\beta I(t)-(\rho +\eta +\alpha )-(\rho +\theta )- \frac{1}{2} \biggl[\sigma _{1}^{2}+\sigma _{2}^{2}+\sigma _{3}^{2} \\ &\quad{}+\sigma _{4}^{2} \biggl( \frac{I(t)}{\varphi (I(t))} \biggr)^{2}+ \sigma _{4}^{2} \biggl(\frac{S(t)}{\varphi (I(t))} \biggr)^{2} \biggr] \\ &:= G\bigl(S(t),I(t)\bigr). \end{aligned}$$

The inequality (4) holds since $\varphi (I(t))\geq 1$. Then,

$$ \begin{aligned} \mathrm{d}V\bigl(S(t),I(t),R(t)\bigr)&\geq G \bigl(S(t),I(t)\bigr)+\sigma _{1}\,\mathrm{d}B_{1}(t)+ \sigma _{2}\,\mathrm{d}B_{2}(t)+\sigma _{3}\,\mathrm{d}B_{3}(t) \\ &\quad{}+\sigma _{4}\frac{S(t)-I(t)}{\varphi (I(t))}\,\mathrm{d}B_{4}(t). \end{aligned} $$

Integrate both sides of $\mathrm{d}V(S(t),I(t),R(t))$ from 0 to *t*, to yield

5 $$\begin{aligned} V\bigl(S(t),I(t),R(t)\bigr)&\geq V\bigl(S(0),I(0),R(0)\bigr)+ \int _{0}^{t}G\bigl(S(s),I(s)\bigr)\, \mathrm{d}s + \sigma _{1}B_{1}(t) \\ &\quad{}+\sigma _{2}B_{2}(t)+\sigma _{3}B_{3}(t)+ \int _{0}^{t}\sigma _{4} \frac{S(s)-I(s)}{\varphi (I(s))}\,\mathrm{d}B_{4}(s). \end{aligned}$$

From the definition of $\tau _{+}$, we have $\lim_{t\rightarrow \tau _{+}}V(S(t),I(t),R(t))=-\infty $. Hence, it follows that (5) satisfies

6 $$\begin{aligned} -\infty &\geq V\bigl(S(0),I(0),R(0)\bigr)+ \int _{0}^{\tau _{+}}G\bigl(S(s),I(s)\bigr)\,\mathrm{d}s + \sigma _{1}B_{1}(\tau _{+})+\sigma _{2}B_{2}(\tau _{+}) \\ &\quad{}+\sigma _{3}B_{3}(\tau _{+})+ \int _{0}^{\tau _{+}}\sigma _{4} \frac{S(s)-I(s)}{\varphi (I(s))}\,\mathrm{d}B_{4}(s) \end{aligned}$$

for $t\rightarrow \tau _{+}$. On the other hand, $V(S(0),I(0),R(0))=\ln (S(0)I(0)R(0))>-\infty $ since $(S(0), I(0),R(0))\in {\mathbb{R}}_{+}^{3}$. Then,

7 $$\begin{aligned} &V\bigl(S(0),I(0),R(0)\bigr)+ \int _{0}^{\tau _{+}}G\bigl(S(s),I(s)\bigr)\,\mathrm{d}s + \sigma _{1}B_{1}(\tau _{+})+\sigma _{2}B_{2}(\tau _{+}) \\ &\quad{}+\sigma _{3}B_{3}(\tau _{+})+ \int _{0}^{\tau _{+}}\sigma _{4} \frac{S(s)-I(s)}{\varphi (I(s))}\,\mathrm{d}B_{4}(s)>-\infty . \end{aligned}$$

Combining (6) with (7), we have

$$ \begin{aligned} -\infty &\geq V\bigl(S(0),I(0),R(0)\bigr)+ \int _{0}^{\tau _{+}}G\bigl(S(s),I(s)\bigr)\,\mathrm{d}s + \sigma _{1}B_{1}(\tau _{+})+\sigma _{2}B_{2}(\tau _{+}) \\ &\quad{}+\sigma _{3}B_{3}(\tau _{+})+ \int _{0}^{\tau _{+}}\sigma _{4} \frac{S(s)-I(s)}{\varphi (I(s))}\,\mathrm{d}B_{4}(s)>-\infty . \end{aligned} $$

Obviously, this is a contradiction. Therefore, $\tau _{+}=+\infty $. The proof is complete. □

## Extinction of a disease

The extinction of a disease has always been a concern. In the deterministic model (2), the disease will be extinct if ${\mathscr{R}}< 1$. That is to say, the disease-free equilibrium $E^{0}(\frac{\Lambda}{\rho},0,0)$ is globally asymptotically stable. However, the condition of disease extinction in model (3) is different from that of the model (2). Define a parameter

$$ {\mathscr{R}}^{s}= \frac{\beta ^{2}}{2\sigma _{4}^{2}(\rho +\eta +\alpha +\frac{1}{2}\sigma _{2}^{2})}.$$

### Theorem 2

*For any initial value* $(S(0),I(0),R(0))\in {\mathbb{R}}_{+}$ *in model* (3), *if* ${\mathscr{R}}^{s}<1$, *then the disease* $I(t)$ *will die out exponentially with probability one*; *that is*,

$$ \limsup_{t\rightarrow +\infty}\frac{\ln I(t)}{t}\leq \biggl( \rho +\eta + \alpha +\frac{1}{2}\sigma _{2}^{2} \biggr) \bigl({\mathscr{R}}^{s}-1\bigr)< 0 \quad \textit{a.s.} $$

### Proof

Through Itô’s formula, we have

$$ \begin{aligned} \mathrm{d}\ln I(t)&= \biggl[\frac{1}{I(t)} \biggl( \frac{\beta S(t)I(t)}{\varphi (I(t))}-(\rho +\eta +\alpha )I(t) \biggr)- \frac{1}{2}\sigma _{2}^{2}- \frac{1}{2}\sigma _{4}^{2} \biggl( \frac{S(t)}{\varphi (I(t))} \biggr)^{2} \biggr]\,\mathrm{d}t \\ &\quad{}+\sigma _{2}\,\mathrm{d}B_{2}(t)+\sigma _{4}\frac{S(t)}{\varphi (I(t))}\,\mathrm{d}B_{4}(t). \end{aligned} $$

Integrating both sides of the above equation from 0 to *t* leads to

$$ \begin{aligned} \ln I(t)&= \ln I(0)+ \int _{0}^{t} \biggl[ \frac{\beta S(s)}{\varphi (I(s))}-( \rho +\eta +\alpha )-\frac{1}{2} \sigma _{2}^{2}- \frac{1}{2}\sigma _{4}^{2} \biggl( \frac{S(s)}{\varphi (I(s))} \biggr)^{2} \biggr]\,\mathrm{d}s \\ &\quad{}+\sigma _{2}B_{2}(t)+ \int _{0}^{t}\sigma _{4} \frac{S(s)}{\varphi (I(s))}\,\mathrm{d}B_{4}(s). \end{aligned} $$

Denote $Q(t)=\int _{0}^{t}\sigma _{4}\frac{S(s)}{\varphi (I(s))}\,\mathrm{d}B_{4}(s)$. Obviously, $Q(t)$ is a continuous local martingale, and the quadratic variation satisfies

$$ \bigl\langle Q(t),Q(t)\bigr\rangle =\sigma _{4}^{2} \int _{0}^{t} \biggl( \frac{S(s)}{\varphi (I(s))} \biggr)^{2}\,\mathrm{d}s.$$

Based on Lemma 1, take $m_{k}=m<1$, $n_{k}=k$ and $v=2$. Hence, it follows that

8 $$ Q(t)\leq \frac{1}{2}m\sigma _{4}^{2} \int _{0}^{t} \biggl( \frac{S(s)}{\varphi (I(s))} \biggr)^{2}\,\mathrm{d}s+\frac{2\ln k}{m}, \quad 0\leq t\leq k. $$

Then,

$$ \begin{aligned} \ln I(t)&\leq \ln I(0)+ \int _{0}^{t} \biggl[ \frac{\beta S(s)}{\varphi (I(s))}- \biggl(\rho +\eta +\alpha + \frac{1}{2}\sigma _{2}^{2} \biggr) \\ &\quad{}-\frac{1}{2}(1-m)\sigma _{4}^{2} \biggl( \frac{S(s)}{\varphi (I(s))} \biggr)^{2}\biggr]\,\mathrm{d}s+\sigma _{2}B_{2}(t)+ \frac{2}{m}\ln k. \end{aligned} $$

Through calculation, we have

$$ \begin{aligned} &\frac{\beta S}{\varphi (I)}- \biggl(\rho +\eta +\alpha + \frac{1}{2} \sigma _{2}^{2} \biggr)- \frac{1}{2}(1-m)\sigma _{4}^{2} \biggl( \frac{S}{\varphi (I)} \biggr)^{2} \\ &\quad = -\frac{1}{2}(1-m)\sigma _{4}^{2} \biggl( \frac{S}{\varphi (I)}- \frac{\beta}{(1-m)\sigma _{4}^{2}} \biggr)^{2}+ \frac{\beta ^{2}}{2(1-m)\sigma _{4}^{2}} \\ &\quad \quad{}- \biggl(\rho +\eta +\alpha +\frac{1}{2}\sigma _{2}^{2} \biggr) \\ &\quad \leq \frac{\beta ^{2}}{2(1-m)\sigma _{4}^{2}}- \biggl(\rho +\eta + \alpha +\frac{1}{2} \sigma _{2}^{2} \biggr) \\ &\quad = \biggl(\rho +\eta +\alpha +\frac{1}{2}\sigma _{2}^{2} \biggr) \biggl(\frac{1}{1-m}{\mathscr{R}}^{s}-1 \biggr). \end{aligned} $$

Hence,

$$ \begin{aligned} \ln I(t)&\leq \ln I(0)+ \int _{0}^{t} \biggl(\rho +\eta +\alpha + \frac{1}{2}\sigma _{2}^{2} \biggr) \biggl( \frac{1}{1-m}{\mathscr{R}}^{s}-1 \biggr)\,\mathrm{d}s \\ &\quad{}+\sigma _{2}B_{2}(t)+\frac{2}{m}\ln k \\ &= \ln I(0)+ \biggl(\rho +\eta +\alpha +\frac{1}{2}\sigma _{2}^{2} \biggr) \biggl(\frac{1}{1-m}{\mathscr{R}}^{s}-1 \biggr)t \\ &\quad{}+\sigma _{2}B_{2}(t)+\frac{2}{m}\ln k. \end{aligned} $$

Dividing the inequality by *t* ($t\in [k-1,k]$), we obtain

$$ \begin{aligned} \frac{\ln I(t)}{t}&\leq \frac{\ln I(0)}{t}+ \biggl(\rho +\eta + \alpha +\frac{1}{2}\sigma _{2}^{2} \biggr) \biggl(\frac{1}{1-m}{ \mathscr{R}}^{s}-1 \biggr) \\ &\quad{}+\frac{\sigma _{2}B_{2}(t)}{t}+\frac{2}{m}\frac{\ln k}{t}. \end{aligned} $$

From the strong law of large numbers, when $k\rightarrow +\infty $, i.e., $t\rightarrow +\infty $, this yields

$$ \limsup_{t\rightarrow \infty}\frac{\ln I(t)}{t}\leq \biggl( \rho +\eta + \alpha +\frac{1}{2}\sigma _{2}^{2} \biggr) \biggl( \frac{1}{1-m}{\mathscr{R}}^{s}-1 \biggr). $$

The desired inequality holds by letting $m\rightarrow 0$. The proof is complete. □

### Example 1

Let $\varphi (I)=1+I^{2}$ in the models (2) and (3).

**Figure 1:**
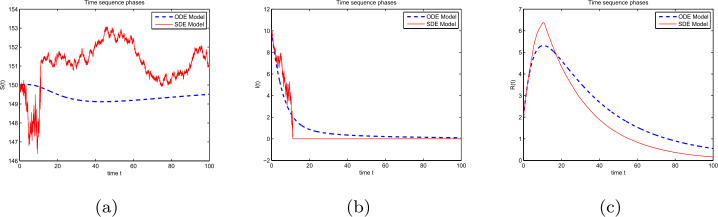
Dynamical curves of compartments: (**a**) *S*, (**b**) *I*, and (**c**) *R* under ${\mathscr{R}}<1$ and ${\mathscr{R}}^{s}<1$
(ii) Let $\beta =0.009$; other parameters and initial values are the same as those in (i). After direct calculation, ${\mathscr{R}}=9>1$ and ${\mathscr{R}}^{s}=0.1775<1$. Figure 2 shows that the disease will become extinct in a random environment but persist in a deterministic environment.

(i) Take $(S(0),I(0),R(0)) = (150,10,2)$, $(\Lambda , \beta , \rho , p, \eta , \theta , \alpha ) = (6, 0.0009, 0.04, 0.97, 0.1, 0.001, 0.01)$, and $(\sigma _{1}, \sigma _{2}, \sigma _{3}, \sigma _{4}) = (0.0016, 0.0032, 0.0022, 0.039)$. After direct calculation, ${\mathscr{R}}=0.9<1$ and ${\mathscr{R}}^{s}=0.0018<1$. Figure 1 shows that the disease will become extinct in both a random and deterministic environment.

**Figure 2:**
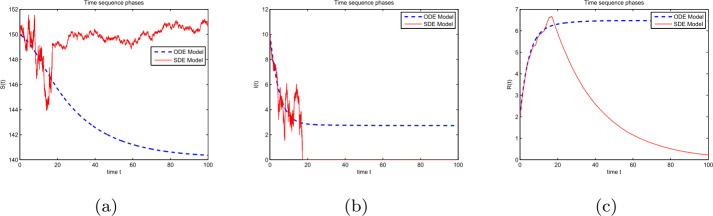
Dynamical curves of compartments: (**a**) *S*, (**b**) *I*, and (**c**) *R* under ${\mathscr{R}}>1$ and ${\mathscr{R}}^{s}<1$

## Asymptotic behavior around the disease-free equilibrium

In the model (2), the disease-free equilibrium $E^{0}(\frac{\Lambda}{\rho},0,0)$ is globally asymptotically stable when ${\mathscr{R}}<1$. However, $E^{0}$ is no longer the equilibrium of model (3) due to the stochastic perturbations. Thus, it is interesting to study the asymptotic behavior of the solution of model (3) around $E^{0}$.

### Theorem 3

*If* ${\mathscr{R}}\leq 1$ *and* $\sigma _{1}^{2}<\frac{\rho}{2}$, $\sigma _{2}^{2}<\rho +2\alpha +2p \eta -\frac{\eta ^{2}p^{2}}{\rho}$, $\sigma _{3}^{2}<\rho +2\theta - \frac{2\theta ^{2}}{\rho}$, *then the solution of model* (3) *satisfies*

$$ \limsup_{t\rightarrow \infty}\frac{1}{t}{\mathbb{E}} \int _{0}^{t} \biggl[ \biggl(S(s)- \frac{\Lambda}{\rho} \biggr)^{2}+I^{2}(s)+R^{2}(s) \biggr]\,\mathrm{d}s\leq \frac{\sigma _{1}^{2}\Lambda ^{2}}{M\rho ^{2}} $$

*for any given initial value* $(S(0),I(0),R(0))\in \mathbb{R}_{+}^{3}$, *where*

$$ M=\min \biggl\{ \frac{\rho}{2}-\sigma _{1}^{2}, \frac{\rho}{2}+\alpha +p \eta -\frac{\eta ^{2} p^{2}}{2\rho}-\frac{\sigma _{2}^{2}}{2}, \frac{\rho}{2}+\theta -\frac{\sigma _{3}^{2}}{2}- \frac{\theta ^{2}}{\rho} \biggr\} . $$

### Proof

Let $x=S(t)-\Lambda /\rho $, $y=I(t)$ and $z=R(t) $. Substituting these variables into the model (3), one can obtain the following equations

$$ \textstyle\begin{cases} \mathrm{d}x= [\Lambda -\rho (x+\frac{\Lambda}{\rho} )- \frac{\beta (x+\Lambda /\rho )}{\varphi (y)}y+(1-p)\eta y+ \theta z ]\,\mathrm{d}t \\ \hphantom{\mathrm{d}x={}}{} +\sigma _{1} (x+\frac{\Lambda}{\rho} )\,\mathrm{d}B_{1}(t)- \sigma _{4}\frac{x+\Lambda /\rho}{\varphi (y)}y\,\mathrm{d}B_{4}(t), \\ \mathrm{d}y= [ \frac{\beta (x+\Lambda /\rho )}{\varphi (y)}y-(\rho + \eta +\alpha )y ]\,\mathrm{d}t+\sigma _{2}y\,\mathrm{d}B_{2}(t)+\sigma _{4} \frac{x+\Lambda /\rho}{\varphi (y)}y\,\mathrm{d}B_{4}(t), \\ \mathrm{d}z=[p\eta y-(\rho +\theta )z]\,\mathrm{d}t+\sigma _{3} z\,\mathrm{d}B_{3}(t). \end{cases} $$

Denote $\Phi _{1}=\frac{1}{2}(x+y)^{2}$, $\Phi _{2}=y$ and $\Phi _{3}=\frac{1}{2}z^{2}$. Define a function $\Phi =\Phi _{1}+\frac{2\rho +\alpha +p\eta}{\beta}\Phi _{2}+\Phi _{3}$. Then,

$$ \mathrm{d}\Phi =\mathrm{d}\Phi _{1}+\frac{2\rho +\alpha +p\eta}{\beta}\,\mathrm{d} \Phi _{2}+\mathrm{d}\Phi _{3}, $$

where

$$ \begin{aligned} \mathrm{d}\Phi _{1}&= {\mathcal {L}}\Phi _{1}\,\mathrm{d}t+(x+y) \biggl[\sigma _{1} \biggl(x+ \frac{\Lambda}{\rho} \biggr) \biggr]\,\mathrm{d}B_{1}(t)+(x+y) \sigma _{2}y\,\mathrm{d}B_{2}(t), \\ \mathrm{d}\Phi _{2}&= {\mathcal {L}}\Phi _{2}\,\mathrm{d}t+ \sigma _{2}y\,\mathrm{d}B_{2}(t)+\sigma _{4} \frac{x+\Lambda /\rho}{\varphi (y)}y\,\mathrm{d}B_{4}(t),\qquad \mathrm{d}\Phi _{3}={\mathcal {L}}\Phi _{3}\,\mathrm{d}t+ \sigma _{3}z^{2}\,\mathrm{d}B_{3}(t). \end{aligned} $$

Through calculation, we have

9 $$\begin{aligned} {\mathcal {L}}\Phi _{1}&= (x+y) \biggl[-\rho x- \frac{\beta (x+\Lambda /\rho )}{\varphi (y)}y+(1-p)\eta y+ \theta z \biggr] \\ &\quad{}+(x+y) \biggl[ \frac{\beta (x+\Lambda /\rho )}{\varphi (y)}y-(\rho + \eta +\alpha )y \biggr]+ \frac{\sigma _{1}^{2} (x+\Lambda /\rho )^{2}+\sigma _{2}^{2}y^{2}}{2} \\ &= (x+y)\bigl[-\rho x-(\rho +\alpha +p\eta )y+\theta z\bigr]+ \frac{\sigma _{1}^{2} (x+\Lambda /\rho )^{2}+\sigma _{2}^{2}y^{2}}{2} \\ &= -\rho x^{2}-(\rho +\alpha +p\eta )y^{2}-(2 \rho +\alpha +p\eta )xy+ \theta xz+\theta yz \\ &\quad{}+ \frac{\sigma _{1}^{2} (x+\Lambda /\rho )^{2}+\sigma _{2}^{2}y^{2}}{2} \\ &= -\rho x^{2}-(\rho +\alpha +p\eta )y^{2}-(2 \rho +\alpha +p\eta )xy+ \frac{\theta}{\sqrt{\rho}}z\sqrt{\rho}x+\frac{\theta}{\sqrt{\rho}} z \sqrt{\rho}y \\ &\quad{}+ \frac{\sigma _{1}^{2} (x+\Lambda /\rho )^{2}+\sigma _{2}^{2}y^{2}}{2} \\ &\leq -\rho x^{2}-(\rho +\alpha +p\eta )y^{2}-(2 \rho +\alpha +p\eta )xy+ \frac{\theta ^{2}}{2\rho}z^{2}+ \frac{\rho}{2}x^{2}+ \frac{\theta ^{2}}{2\rho}z^{2} \\ &\quad{}+\frac{\rho}{2}y^{2}+\sigma _{1}^{2}x^{2}+\sigma _{1}^{2} \biggl( \frac{\Lambda}{\rho} \biggr)^{2}+\frac{\sigma _{2}^{2}y^{2}}{2} \end{aligned}$$

10 $$\begin{aligned} &= - \biggl(\frac{\rho}{2}-\sigma _{1}^{2} \biggr)x^{2}- \biggl( \frac{\rho}{2}+\alpha +p\eta - \frac{\sigma _{2}^{2}}{2} \biggr)y^{2}+ \frac{\theta ^{2}}{\rho}z^{2}+ \sigma _{1}^{2} \frac{\Lambda ^{2}}{\rho ^{2}} \\ &\quad{}-(2\rho +\alpha +p\eta )xy, \\ {\mathcal {L}}\Phi _{2} &= \frac{\beta (x+\Lambda /\rho )}{\varphi (y)}y-(\rho + \eta + \alpha )y \leq \beta xy+ \biggl(\frac{\beta \Lambda}{\rho}-( \rho +\eta +\alpha ) \biggr)y \leq \beta xy, \end{aligned}$$

11 $$\begin{aligned} {\mathcal {L}}\Phi _{3}&= z\bigl(p\eta y-(\rho +\theta )z\bigr)+ \frac{1}{2}\sigma _{3}^{2}z^{2}= p \eta yz- \biggl(\rho +\theta -\frac{\sigma _{3}^{2}}{2} \biggr)z^{2} \\ &= \frac{\eta p}{\sqrt{\rho}}y\sqrt{\rho}z- \biggl(\rho +\theta - \frac{\sigma _{3}^{2}}{2} \biggr)z^{2}\leq \frac{\eta ^{2} p^{2}}{2\rho}y^{2}- \biggl(\frac{\rho}{2}+\theta - \frac{\sigma _{3}^{2}}{2} \biggr)z^{2}. \end{aligned}$$

The inequalities (9) and (11) follow from the fact that $ab\leq (a^{2}+b^{2})/2$ and $(a+b)^{2}\leq 2a^{2}+2b^{2}$ for any $a,b\in \mathbb{R}$. The inequality (10) holds due to ${\mathscr{R}}< 1$. Combining these equations, we have

12 $$\begin{aligned} {\mathcal {L}}\Phi &\leq - \biggl(\frac{\rho}{2}-\sigma _{1}^{2} \biggr)x^{2}- \biggl( \frac{\rho}{2}+\alpha +p\eta -\frac{\eta ^{2} p^{2}}{2\rho}- \frac{\sigma _{2}^{2}}{2} \biggr)y^{2} \\ &\quad{}- \biggl(\frac{\rho}{2}+\theta -\frac{\sigma _{3}^{2}}{2}- \frac{\theta ^{2}}{\rho} \biggr)z^{2}+\sigma _{1}^{2} \frac{\Lambda ^{2}}{\rho ^{2}}. \end{aligned}$$

Hence,

$$ \begin{aligned} \mathrm{d}\Phi &\leq \biggl[- \biggl( \frac{\rho}{2}-\sigma _{1}^{2} \biggr)x^{2}- \biggl(\frac{\rho}{2}+\alpha +p\eta - \frac{\eta ^{2} p^{2}}{2\rho}-\frac{\sigma _{2}^{2}}{2} \biggr)y^{2}- \biggl( \frac{\rho}{2}+\theta -\frac{\sigma _{3}^{2}}{2}- \frac{\theta ^{2}}{\rho} \biggr)z^{2} \\ &\quad{}+\sigma _{1}^{2}\frac{\Lambda ^{2}}{\rho ^{2}} \biggr] \,\mathrm{d}t+(x+y) \biggl[\sigma _{1} \biggl(x+\frac{\Lambda}{\rho} \biggr) \biggr]\,\mathrm{d}B_{1}(t) \\ &\quad{}+\sigma _{2}y \biggl(x+y+\frac{2\rho +\alpha +p\eta}{\beta} \biggr) \,\mathrm{d}B_{2}(t)+\sigma _{3}z^{2}\,\mathrm{d}B_{3}(t). \end{aligned} $$

Integrating both sides of the inequality from 0 to *t*, and taking the expectation yields

$$ \begin{aligned} 0&\leq {\mathbb{E}}\bigl[\Phi (t)\bigr] \\ &\leq {\mathbb{E}}\bigl[\Phi (0)\bigr]+{\mathbb{E}} \int _{0}^{t} \biggl[- \biggl( \frac{\rho}{2}-\sigma _{1}^{2} \biggr) x^{2}(s)- \biggl(\frac{\rho}{2}+ \alpha +p\eta - \frac{\eta ^{2} p^{2}}{2\rho}- \frac{\sigma _{2}^{2}}{2} \biggr) y^{2}(s) \\ &\quad{}- \biggl(\frac{\rho}{2}+\theta -\frac{\sigma _{3}^{2}}{2}- \frac{\theta ^{2}}{\rho} \biggr) z^{2}(s)+\sigma _{1}^{2} \frac{\Lambda ^{2}}{\rho ^{2}} \biggr]\,\mathrm{d}s, \end{aligned} $$

which implies

$$ \begin{aligned} &{\mathbb{E}} \int _{0}^{t} \biggl[ \biggl( \frac{\rho}{2}-\sigma _{1}^{2} \biggr)x^{2}(s)+ \biggl(\frac{\rho}{2}+\alpha +p\eta - \frac{\eta ^{2} p^{2}}{2\rho}-\frac{\sigma _{2}^{2}}{2} \biggr)y^{2}(s) \\ &\quad{}+ \biggl(\frac{\rho}{2}+\theta -\frac{\sigma _{3}^{2}}{2}- \frac{\theta ^{2}}{\rho} \biggr)z^{2}(s)+\sigma _{1}^{2} \frac{\Lambda ^{2}}{\rho ^{2}} \biggr]\,\mathrm{d}s\leq {\mathbb{E}}\bigl[\Phi (0)\bigr]+ \sigma _{1}^{2}\frac{\Lambda ^{2}}{\rho ^{2}}t. \end{aligned} $$

Therefore,

$$ \begin{aligned} &\limsup_{t\rightarrow \infty} \frac{1}{t}{\mathbb{E}} \int _{0}^{t} \biggl[ \biggl( \frac{\rho}{2}-\sigma _{1}^{2} \biggr)x^{2}(s)+ \biggl( \frac{\rho}{2}+\alpha +p\eta - \frac{\eta ^{2} p^{2}}{2\rho}- \frac{\sigma _{2}^{2}}{2} \biggr)y^{2}(s) \\ &\quad{}+ \biggl(\frac{\rho}{2}+\theta -\frac{\sigma _{3}^{2}}{2}- \frac{\theta ^{2}}{\rho} \biggr)z^{2}(s)+\sigma _{1}^{2} \frac{\Lambda ^{2}}{\rho ^{2}} \biggr]\,\mathrm{d}s\leq \sigma _{1}^{2} \frac{\Lambda ^{2}}{\rho ^{2}}. \end{aligned} $$

Then,

$$ \limsup_{t\rightarrow \infty}\frac{1}{t}{\mathbb{E}} \int _{0}^{t}\bigl(x^{2}(s)+y^{2}(s)+z^{2}(s) \bigr)\,\mathrm{d}s\leq \frac{\sigma _{1}^{2}\Lambda ^{2}}{M\rho ^{2}}, $$

i.e.,

$$ \limsup_{t\rightarrow \infty}\frac{1}{t}{\mathbb{E}} \int _{0}^{t} \biggl[ \biggl(S(s)- \frac{\Lambda}{\rho} \biggr)^{2}+ I^{2}(s)+R^{2}(s) \biggr]\,\mathrm{d}s\leq \frac{\sigma _{1}^{2}\Lambda ^{2}}{M\rho ^{2}}. $$

The result follows. □

### Remark 1

Theorem 3 shows that if ${\mathscr{R}}\leq 1$ and $\sigma _{i}$ ($i=1,2,3$) satisfy certain conditions, the solution $(S(t),I(t),R(t))$ of model (3) oscillates around $E^{0}$, and the intensity of the oscillation is determined by $\sigma _{i}$ ($i=1,2,3$). Further, when $\sigma _{i}$ ($i=1,2,3$) decrease, the solution $(S(t),I(t),R(t))$ of model (3) is close to the disease-free equilibrium $E^{0}$. If $\sigma _{1}=0$, (12) is simplified as

$$ \begin{aligned} {\mathcal {L}}\Phi &\leq -\frac{\rho}{2} \biggl(S-\frac{\Lambda}{\rho} \biggr)^{2}- \biggl( \frac{\rho}{2}+\alpha +p\eta - \frac{\eta ^{2} p^{2}}{2\rho}-\frac{\sigma _{2}^{2}}{2} \biggr)I^{2} \\ &\quad{}- \biggl(\frac{\rho}{2}+\theta -\frac{\sigma _{3}^{2}}{2}- \frac{\theta ^{2}}{\rho} \biggr)R^{2}, \end{aligned} $$

which is negative-definite when $\sigma _{2}^{2}<\rho +2\alpha +2p\eta -\frac{\eta ^{2}p^{2}}{\rho}$, $\sigma _{3}^{2}<\rho +2\rho -\frac{2\rho ^{2}}{\rho}$. Therefore, the disease-free equilibrium $E^{0}$ of model (3) is stochastically asymptotically stable.

### Example 2

**Figure 3:**
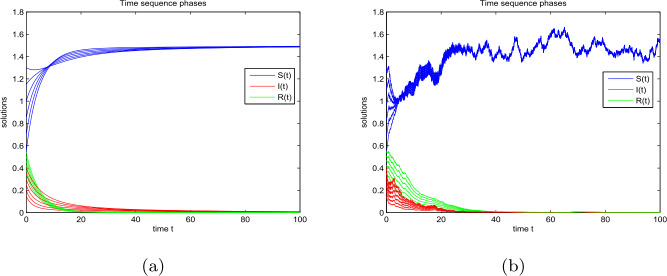
(**a**) Time-series phases of solutions ($S(t)$, $I(t)$, $R(t)$) for model (2); (**b**) Time-series phases of solutions ($S(t)$, $I(t)$, $R(t)$) for model (3). The parameters are taken from Example 2

Assume that $(S(0),I(0),R(0)) = (0.4,0.1,0.3)$, $(\Lambda , \beta , \rho , p, \eta , \theta , \alpha )= (0.3, 0.4, 0.2, 0.97, 0.31, 0.01, 0.1)$, and $(\sigma _{1}, \sigma _{2}, \sigma _{3}, \sigma _{4})= (0.03, 0.08, 0.04, 0.12)$. After calculation, one can see that ${\mathscr{R}}=0.9836<1$ and $\sigma _{1}^{2}<\frac{\rho}{2}$, $\sigma _{2}^{2}<\rho +2\alpha +2p \eta -\frac{\eta ^{2}p^{2}}{\rho}$, $\sigma _{3}^{2}<\rho +2\theta - \frac{2\theta ^{2}}{\rho}$, satisfying the conditions of Theorem 3. Figure 3 shows the trajectories of models (2) and (3). The disease-free equilibrium $E^{0}(1.5,0,0)$ is global asymptotically stable. The solution of model (3) is around the solution of model (2).

## Existence of the stationary distribution

In this section, we mainly study two properties: (i) the asymptotic behavior of the solution of the model (3) around the endemic equilibrium $E^{*}(S^{*}, I^{*}, R^{*})$ of the model (2), and (ii) the existence and uniqueness of the stationary distribution of the solution for the model (3). Denote

$$\begin{aligned}& \kappa _{1} = \frac{\rho}{2}-\sigma _{1}^{2}, \qquad \kappa _{2}= \frac{\rho}{2}+\alpha +p\eta - \frac{p^{2}\eta ^{2}}{2\rho}-\sigma _{2}^{2}, \qquad \kappa _{3}=\frac{\rho}{2}+\theta -\frac{\theta ^{2}}{\rho}- \sigma _{3}^{2}, \\& W = \sigma _{1}^{2}S^{*2}+\sigma _{2}^{2} \biggl(I^{*2}+ \frac{2\rho +\alpha +p\eta}{2\beta}I^{*}\varphi \bigl(I^{*}\bigr) \biggr)+ \sigma _{3}^{2}R^{*2}. \end{aligned}$$

### Theorem 4

*If* ${\mathscr{R}}>1$ *and* $0< W<\min \{\kappa _{1}S^{*2},\kappa _{2}I^{*2},\kappa _{3}R^{*2}\}$, *then*

13 $$ \limsup_{t\rightarrow \infty}\frac{1}{t}{\mathbb{E}} \int _{0}^{t}\bigl[ \kappa _{1} \bigl(S(s)-S^{*}\bigr)^{2}+\kappa _{2} \bigl(I(s)-I^{*}\bigr)^{2}+\kappa _{3} \bigl(R(s)-R^{*}\bigr)^{2}\bigr]\,\mathrm{d}s\leq W. $$

*Further*, *there is a unique stationary distribution* *π* *for the solution of model* (3).

### Proof

We first prove the inequality (13). Since ${\mathscr{R}}>1$, model (2) has a unique endemic equilibrium $E^{*}(S^{*},I^{*},R^{*})$ satisfying

$$ p\eta I^{*}=(\rho +\theta )R^{*},\qquad \frac{\beta S^{*}}{\varphi (I^{*})}=\rho +\eta +\alpha ,\qquad \Lambda =\rho S^{*}+ \frac{\beta S^{*}I^{*}}{\varphi (I^{*})}-(1-p) \eta I^{*}-\theta R^{*}. $$

Let $\Psi _{1}=\frac{1}{2}(S+I-S^{*}-I^{*})^{2}$, $\Psi _{2}=I-I^{*}-I^{*} \ln \frac{I}{I^{*}}$ and $\Psi _{3}=\frac{1}{2}(R-R^{*})^{2}$. Define a function

$$ \Psi =\Psi _{1}+\frac{2\rho +\alpha +p\eta}{\beta}\varphi \bigl(I^{*} \bigr) \Psi _{2}+\Psi _{3}. $$

Then,

$$ \mathrm{d}\Psi =\mathrm{d}\Psi _{1}+\frac{2\rho +\alpha +p\eta}{\beta} \varphi \bigl(I^{*}\bigr)\,\mathrm{d}\Psi _{2}+\mathrm{d}\Psi _{3}. $$

By Itô’s formula, we have

$$ \begin{aligned} &\mathrm{d}\Psi _{1} ={\mathcal {L}}\Psi _{1}\,\mathrm{d}t+\bigl(S+I-S^{*}-I^{*}\bigr) \bigl( \sigma _{1}S\,\mathrm{d}B_{1}(t)+\sigma _{2}I\,\mathrm{d}B_{2}(t)\bigr), \\ &\mathrm{d}\Psi _{2} ={\mathcal {L}}\Psi _{2}\,\mathrm{d}t+ \bigl(I-I^{*}\bigr)\sigma _{2} \,\mathrm{d}B_{2}(t)+\bigl(I-I^{*}\bigr)\sigma _{4} \frac{SI}{\varphi (I)}\,\mathrm{d}B_{4}(t), \\ &\mathrm{d}\Psi _{3} ={\mathcal {L}}\Psi _{3}\,\mathrm{d}t+ \bigl(R-R^{*}\bigr)R\sigma _{3} \,\mathrm{d}B_{3}(t), \end{aligned} $$

where

14 $$\begin{aligned} {\mathcal {L}}\Psi _{1}&= \bigl(S+I-S^{*}-I^{*} \bigr)\bigl[\Lambda -\rho S-(\rho + \alpha +p\eta )I+\theta R\bigr]+ \frac{\sigma _{1}^{2}}{2}S^{2}+ \frac{\sigma _{2}^{2}}{2}I^{2} \\ &= \bigl(S+I-S^{*}-I^{*}\bigr) \bigl[\rho S^{*}+(\rho +\alpha +p\eta )I^{*}- \theta R^{*}-\rho S-(\rho +\alpha +p\eta )I+\theta R \bigr] \\ &\quad{}+\frac{\sigma _{1}^{2}}{2}\bigl(S-S^{*}+S^{*} \bigr)^{2}+ \frac{\sigma _{2}^{2}}{2}\bigl(I-I^{*}+I^{*} \bigr)^{2} \\ &\leq \bigl(S-S^{*}+I-I^{*}\bigr)\bigl[-\rho \bigl(S-S^{*}\bigr)-(\rho +\alpha +p\eta ) \bigl(I-I^{*} \bigr)+ \theta \bigl(R-R^{*}\bigr)\bigr] \\ &\quad{}+\sigma _{1}^{2} \bigl(S-S^{*}\bigr)^{2}+\sigma _{1}^{2}S^{*2}+ \sigma _{2}^{2}\bigl(I-I^{*} \bigr)^{2}+ \sigma _{2}^{2}I^{*2} \end{aligned}$$

15 $$\begin{aligned} &= -\bigl(\rho -\sigma _{1}^{2}\bigr) \bigl(S-S^{*}\bigr)^{2}-\bigl(\rho +\alpha +p\eta -\sigma _{2}^{2}\bigr) \bigl(I-I^{*} \bigr)^{2}-(2 \rho +\alpha +p\eta ) \\ &\quad{}\times \bigl(S-S^{*}\bigr) \bigl(I-I^{*}\bigr)+ \frac{\theta}{\sqrt{\rho}}\bigl(R-R^{*}\bigr)\sqrt{ \rho} \bigl(S-S^{*}\bigr) \\ &\quad{}+\frac{\theta}{\sqrt{\rho}}\bigl(R-R^{*}\bigr)\sqrt{\rho} \bigl(I-I^{*}\bigr)+\sigma _{1}^{2}S^{*2}+ \sigma _{2}^{2}I^{*2} \\ &\leq -\bigl(\rho -\sigma _{1}^{2}\bigr) \bigl(S-S^{*}\bigr)^{2}-\bigl(\rho +\alpha +p\eta - \sigma _{2}^{2}\bigr) \bigl(I-I^{*} \bigr)^{2}-(2\rho +\alpha +p\eta ) \\ &\quad{}\times \bigl(S-S^{*}\bigr) \bigl(I-I^{*}\bigr)+ \frac{\theta ^{2}}{2\rho}\bigl(R-R^{*}\bigr)^{2}+ \frac{\rho}{2}\bigl(I-I^{*}\bigr)^{2}+ \frac{\theta ^{2}}{2\rho}\bigl(R-R^{*}\bigr)^{2} \\ &\quad{}+\frac{\rho}{2}\bigl(S-S^{*}\bigr)+\sigma _{1}^{2}S^{*2}+\sigma _{2}^{2}I^{*2} \end{aligned}$$

16 $$\begin{aligned} &= - \biggl(\frac{\rho}{2}-\sigma _{1}^{2} \biggr) \bigl(S-S^{*}\bigr)^{2}- \biggl( \frac{\rho}{2}+\alpha +p\eta -\sigma _{2}^{2} \biggr) \bigl(I-I^{*}\bigr)^{2}+ \frac{\theta ^{2}}{\rho} \bigl(R-R^{*}\bigr)^{2} \\ &\quad {} -(2\rho +\alpha +p\eta ) \bigl(S-S^{*}\bigr) \bigl(I-I^{*}\bigr)+\sigma _{1}^{2}S^{*2}+ \sigma _{2}^{2}I^{*2}, \\ {\mathcal {L}}\Psi _{2}&= \biggl(1-\frac{I^{*}}{I} \biggr) \biggl[ \frac{\beta SI}{\varphi (I)}-(\rho +\eta +\alpha )I \biggr]+ \frac{1}{2}I^{*}\sigma _{2}^{2} \\ &= \bigl(I-I^{*}\bigr) \biggl[- \frac{\beta S(\varphi (I)-\varphi (I^{*}))}{\varphi (I)\varphi (I^{*})}+ \frac{\beta (S-S^{*})}{\varphi (I^{*})} \biggr]+\frac{1}{2}I^{*} \sigma _{2}^{2} \\ &\leq \frac{\beta (S-S^{*})(I-I^{*})}{\varphi (I^{*})}+\frac{1}{2}I^{*} \sigma _{2}^{2}, \end{aligned}$$

17 $$\begin{aligned} {\mathcal {L}}\Psi _{3}&= \bigl(R-R^{*}\bigr)\bigl[p \eta I-(\rho +\theta )R\bigr]+ \frac{1}{2}\sigma _{3}^{2}R^{2} \\ &= \bigl(R-R^{*}\bigr)\bigl[-p\eta I^{*}+(\rho + \theta )R^{*}+p\eta I-(\rho +\theta ) R\bigr]+\frac{1}{2} \sigma _{3}^{2}\bigl(R-R^{*}+R^{*} \bigr)^{2} \\ &\leq \bigl(R-R^{*}\bigr)\bigl[p\eta \bigl(I-I^{*}\bigr)-(\rho +\theta ) \bigl(R-R^{*}\bigr) \bigr]+\sigma _{3}^{2}\bigl(R-R^{*} \bigr)^{2}+ \sigma _{3}^{2}R^{*2} \end{aligned}$$

18 $$\begin{aligned} &= \sqrt{\rho}\bigl(R-R^{*}\bigr)\frac{p\eta}{\sqrt{\rho}} \bigl(I-I^{*}\bigr)-\bigl(\rho + \theta -\sigma _{3}^{2} \bigr) \bigl(R-R^{*}\bigr)^{2}+\sigma _{3}^{2}R^{*2} \\ &\leq \frac{\rho}{2}\bigl(R-R^{*} \bigr)^{2}+\frac{p^{2}\eta ^{2}}{2\rho}\bigl(I-I^{*} \bigr)^{2}-\bigl( \rho +\theta -\sigma _{3}^{2} \bigr) \bigl(R-R^{*}\bigr)^{2}+\sigma _{3}^{2}R^{*2} \\ &= - \biggl(\frac{\rho}{2}+\theta -\sigma _{3}^{2} \biggr) \bigl(R-R^{*}\bigr)^{2}+ \frac{p^{2}\eta ^{2}}{2\rho} \bigl(I-I^{*}\bigr)^{2}+\sigma _{3}^{2}R^{*2}. \end{aligned}$$

The inequalities (15) and (18) hold because $ab\leq a^{2}/2+b^{2}/2$, (14) and (17) hold because $(a+b)^{2}\leq 2a^{2}+2b^{2}$, while (16) holds because of the fact that $\frac{\beta S(I-I^{*})(\varphi (I)-\varphi (I^{*}))}{\varphi (I)\varphi (I^{*})}>0$. Combined with the above inequalities, one can obtain

19 $$\begin{aligned} {\mathcal {L}}\Psi &= {\mathcal {L}}\Psi _{1}+ \frac{2\rho +\alpha +p\eta}{\beta}\varphi \bigl(I^{*}\bigr){\mathcal {L}}\Psi _{2}+{ \mathcal {L}}\Psi _{3} \\ &\leq - \biggl(\frac{\rho}{2}-\sigma _{1}^{2} \biggr) \bigl(S-S^{*}\bigr)^{2}- \biggl( \frac{\rho}{2}+\alpha +p\eta -\frac{p^{2}\eta ^{2}}{2\rho}- \sigma _{2}^{2} \biggr) \bigl(I-I^{*} \bigr)^{2} \\ &\quad{}- \biggl(\frac{\rho}{2}+\theta -\frac{\theta ^{2}}{\rho}-\sigma _{3}^{2} \biggr) \bigl(R-R^{*} \bigr)^{2}+\sigma _{1}^{2}S^{*2} \\ &\quad{}+\sigma _{2}^{2} \biggl(I^{*2}+ \frac{2\rho +\alpha +p\eta}{2\beta}I^{*} \varphi \bigl(I^{*}\bigr) \biggr)+\sigma _{3}^{2}R^{*2} \\ &= -\kappa _{1}\bigl(S-S^{*} \bigr)^{2}-\kappa _{2}\bigl(I-I^{*} \bigr)^{2}-\kappa _{3}\bigl(R-R^{*} \bigr)^{2}+W. \end{aligned}$$

Note that

$$ \begin{aligned} \mathrm{d}\Psi &= {\mathcal {L}}\Psi \,\mathrm{d}t+ \bigl(S+I-S^{*}-I^{*}\bigr) \bigl(\sigma _{1}S\,\mathrm{d}B_{1}(t)+\sigma _{2}I\,\mathrm{d}B_{2}(t)\bigr) \\ &\quad{}+\frac{2\rho +\alpha +p\eta}{\beta}\varphi \bigl(I^{*}\bigr) \bigl(I-I^{*}\bigr)\sigma _{2} \,\mathrm{d}B_{2}(t)+\bigl(R-R^{*}\bigr)R\sigma _{3}\,\mathrm{d}B_{3}(t). \end{aligned} $$

Integrating both sides of dΨ from 0 to *t*, and taking the expectations, from (19), this yields

$$ \begin{aligned} 0&\leq {\mathbb{E}}\Psi \bigl(S(t),I(t),R(t)\bigr)-{ \mathbb{E}}\Psi \bigl(S(0),I(0),R(0)\bigr) \\ &\leq {\mathbb{E}} \int _{0}^{t}\bigl[-\kappa _{1} \bigl(S(s)-S^{*}\bigr)^{2}-\kappa _{2} \bigl(I(s)-I^{*}\bigr)^{2}- \kappa _{3} \bigl(R(s)-R^{*}\bigr)^{2}\bigr]\,\mathrm{d}s+Wt. \end{aligned} $$

Dividing both sides by *t* and letting $t\rightarrow \infty $, we have

$$ \limsup_{t\rightarrow \infty} \frac{1}{t}{\mathbb{E}} \int _{0}^{t}\bigl[ \kappa _{1} \bigl(S(s)-S^{*}\bigr)^{2}+\kappa _{2} \bigl(I(s)-I^{*}\bigr)^{2}+\kappa _{3} \bigl(R(s)-R^{*}\bigr)^{2}\bigr]\,\mathrm{d}s\leq W. $$

Then, (13) has been proved.

On the other hand, we only prove Assumptions (A.1) and (A.2) for the existence and uniqueness of the stationary distribution. Consider the ellipsoid $-\kappa _{1}(S-S^{*})^{2}-\kappa _{2}(I-I^{*})^{2}-\kappa _{3}(R-R^{*})^{2}+W=0$, i.e.,

$$ \frac{(S-S^{*})^{2}}{ (\sqrt{\frac{W}{\kappa _{1}}} )^{2}}+ \frac{(I-I^{*})^{2}}{ (\sqrt{\frac{W}{\kappa _{2}}} )^{2}}+ \frac{(R-R^{*})^{2}}{ (\sqrt{\frac{W}{\kappa _{3}}} )^{2}}=1. $$

If $S^{*}>\sqrt{W/\kappa _{1}}$, $I^{*}>\sqrt{W/\kappa _{2}}$ and $R^{*}>\sqrt{W/\kappa _{3}}$, i.e., $W<\min \{\kappa _{1}S^{*2}, \kappa _{2}I^{*2}, \kappa _{3}R^{*2}\}$, then the ellipsoid is fully contained in ${\mathbb{R}}_{+}^{3}$. Let *U* be the neighborhood of the ellipsoid such that $\overline{U}\subset {\mathbb{R}}_{+}^{3}$. Thus,

$$ -\kappa _{1}\bigl(S-S^{*}\bigr)^{2}-\kappa _{2}\bigl(I-I^{*}\bigr)^{2}-\kappa _{3}\bigl(R-R^{*}\bigr)^{2}+W< 0$$

for any $(S,I,R)\in {\mathbb{R}}_{+}^{3}\setminus U$, i.e. ${\mathcal {L}}\Psi <0$ for any $(S,I,R)\in {\mathbb{R}}_{+}^{3}\setminus U$. Assumption (A.2) is then satisfied. We rewrite the model (3) as

$$ \begin{aligned} \mathrm{d} \begin{bmatrix} S(t) \\ I(t) \\ R(t) \end{bmatrix} &= \begin{bmatrix} \Lambda -\rho S-\frac{\beta SI}{\varphi (I)}+(1-p)\eta I+\theta R \\ \frac{\beta SI}{\varphi (I)}-(\rho +\eta +\alpha )I \\ p\eta I-(\rho +\theta )R \end{bmatrix} \,\mathrm{d}t \\ &\quad{}+ \begin{bmatrix} \sigma _{1}S&0&0&-\sigma _{4}\frac{SI}{\varphi (I)} \\ 0&\sigma _{2}I&0&\sigma _{4}\frac{SI}{\varphi (I)} \\ 0&0&\sigma _{3}R&0 \end{bmatrix} \begin{bmatrix} \mathrm{d}B_{1}(t) \\ \mathrm{d}B_{2}(t) \\ \mathrm{d}B_{3}(t) \\ \mathrm{d}B_{4}(t) \end{bmatrix} . \end{aligned} $$

The diffusion matrix of model (3) is

$$ A= \begin{bmatrix} \sigma _{1}^{2}S^{2}+\sigma _{4}^{2}\frac{S^{2}I^{2}}{f^{2}(I)}&- \sigma _{4}^{2}\frac{S^{2}I^{2}}{f^{2}(I)}&0 \\ -\sigma _{4}^{2}\frac{S^{2}I^{2}}{f^{2}(I)}&\sigma _{2}^{2}I^{2}+ \sigma _{4}^{2}\frac{S^{2}I^{2}}{f^{2}(I)}&0 \\ 0&0&\sigma _{3}^{2}R^{2} \end{bmatrix} . $$

Suppose $M=\min_{(S,I,R)\in \overline{U}\subset {\mathbb{R}}_{+}^{3}}\{ \sigma _{1}^{2}S^{2},\sigma _{2}^{2}I^{2},\sigma _{3}^{2}R^{2}\}$. Then, for any $(S,I,R)\in \overline{U}$ and $\zeta =(\zeta _{1}, \zeta _{2},\zeta _{3})\in {\mathbb{R}}_{+}^{3}$, we have

$$ \begin{aligned} \sum_{i,j=1}^{3}a_{ij}(S,I,R) \zeta _{i}\zeta _{j}&= \biggl(\sigma _{1}^{2}S^{2}+ \sigma _{4}^{2} \frac{S^{2}I^{2}}{f^{2}(I)} \biggr)\zeta _{1}^{2}-2 \sigma _{4}^{2}\frac{S^{2}I^{2}}{f^{2}(I)}\zeta _{1} \zeta _{2} \\ &\quad{}+ \biggl(\sigma _{2}^{2}I^{2}+\sigma _{4}^{2} \frac{S^{2}I^{2}}{f^{2}(I)} \biggr)\zeta _{2}^{2}+\sigma _{3}^{2}R^{2} \zeta _{3}^{2} \\ &= \sigma _{1}^{2}S^{2}\zeta _{1}^{2}+\sigma _{2}^{2}I^{2} \zeta _{2}^{2}+ \sigma _{3}^{2}R^{2} \zeta _{3}^{2}+\sigma _{4}^{2} \frac{S^{2}I^{2}}{f^{2}(I)}\bigl(\zeta _{1}^{2}-\zeta _{2}^{2}\bigr) \\ &\geq \sigma _{1}^{2}S^{2}\zeta _{1}^{2}+\sigma _{2}^{2}I^{2} \zeta _{2}^{2}+ \sigma _{3}^{2}R^{2} \zeta _{3}^{2}\geq M \vert \zeta \vert ^{2}, \end{aligned} $$

which meets Assumption (A.1). Therefore, model (3) has a unique stationary distribution *π*. □

### Remark 2

Theorem 4 shows that the solution $(S(t),I(t),R(t))$ of model (3) oscillates around $E^{*}$ if ${\mathscr{R}}\geq 1$ when $\sigma _{i}$ ($i=1,2,3$) and some parameters satisfy certain conditions.

### Example 3

**Figure 4:**
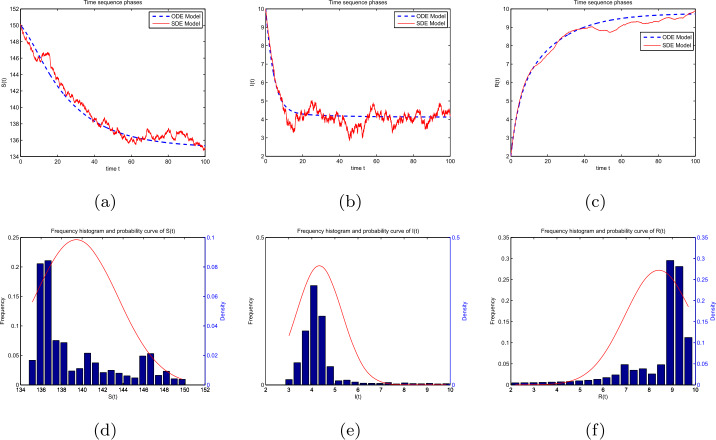
Dynamical curves of compartments: (**a**) *S*, (**b**) *I*, (**c**) *R*. The frequency histograms and marginal density functions of compartments: (**d**) *S*, (**e**) *I*, (**f**) *R*

Let $\varphi (I)=1+I^{2}$, $(S(0),I(0),R(0))= (150,10,2)$, $(\Lambda , \beta , \rho , p, \eta , \theta , \alpha )= (6, 0.02, 0.04, 0.97, 0.1, 0.001, 0.01)$, and $(\sigma _{1}, \sigma _{2}, \sigma _{3}, \sigma _{4})= (0.0016, 0.0032, 0.0022, 0.0085)$, one can obtain that ${\mathscr{R}}=\frac{\Lambda \beta}{\rho (\rho +\eta +\alpha )}=20>1$, $\kappa _{1}=\frac{\rho}{2}-\sigma _{1}^{2}=0.02>0$, $\kappa _{2}= \frac{\rho}{2}+\alpha +p\eta -\frac{p^{2}\eta ^{2}}{2\rho}-\sigma _{2}^{2}=0.0094>0 $, and $\kappa _{3}=\frac{\rho}{2}+\theta -\frac{\theta ^{2}}{\rho}-\sigma _{3}^{2}=0.021>0$. Then, Theorem 4 shows that there exists a unique stationary distribution *π* of model (3). Figures 4(a), (b), and (c) reflect the dynamical population of the susceptible, infective, and recovered individuals in model (2) and model (3), respectively. In Figs. 4(d), (e), and (f), we provide the frequency histogram and corresponding marginal density function curves of compartments *S*, *I*, and *R*, respectively. These two kinds of figures indicate that there exists a unique stationary distribution for model (3).

The following numerical example focuses on the effect of the partial immunization rate *p* on the dynamics of disease transmission.

### Example 4

**Figure 5:**
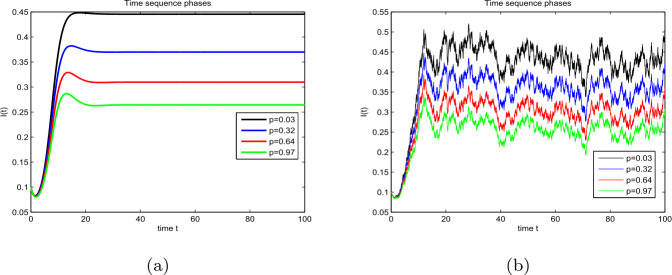
The effect of partial immunity on compartment *I*: (**a**) model (2) and (**b**) model (3)

Let $(S(0),I(0),R(0))= (0.4, 0.1, 0.3)$, $(\Lambda , \beta , \rho , \eta , \theta , \alpha ) = (0.3, 0.9, 0.2, 0.31, 0.01, 0.1)$, and $(\sigma _{1},\sigma _{2},\sigma _{3},\sigma _{4})=(0.03, 0.01, 0.04, 0.1)$. We analyze the effect of partial immunity *p* on the dynamic behavior of model (3). Take $p = 0.03, 0.32, 0.64$, and 0.97. The corresponding curves of $I(t)$ are shown in Figs. 5(a) and (b), respectively. As the value of *p* increases, the growth rate and stability level of $I(t)$ will decrease. This indicates that a large partial immunity rate can better control the epidemic than a small one. Thus, it is effective to increase the immunity rate and control the outbreak of the disease.

**Figure 6:**
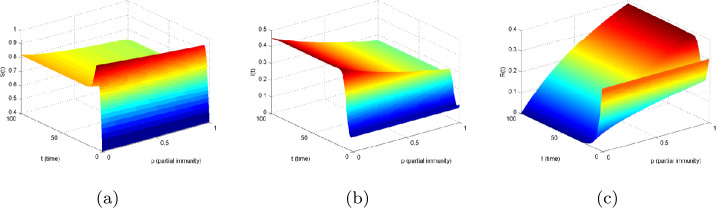
The effect of partial immunity *p* with respect to time *t* for compartments: (**a**) *S*, (**b**) *I*, and (**c**) *R* in model (2)

**Figure 7:**
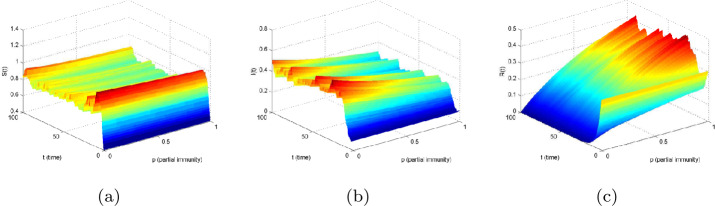
The effect of partial immunity *p* with respect to time *t* for compartments: (**a**) *S*, (**b**) *I*, and (**c**) *R* in model (3)

Based on the models (2) and (3), Figs. 6 and 7 show the sensitivity of partial immunity rate on $S(t)$, $I(t)$, and $R(t)$ in three-dimensional changes in time, respectively. As the increases of *p* in $[0,1]$, the value of $S(t)$ becomes smaller, and $I(t)$ reduces faster than others. However, $R(t)$ increases with the increase of *p*.

## Conclusion

In this paper, we propose a stochastic SIRS model with partial immunity and noninear incidence. Through a theoretical derivation, the following results are obtained for the kind of models: (i) By constructing a suitable function, the SIRS model has a unique global positive solution starting from the positive initial value (see Theorem 1). (ii) If ${\mathscr{R}}^{s}<1$, the disease will become extinct under the stochastic system (see Theorem 2). The result reveals that the large stochastic perturbations may lead to disease extinction. (iii) If ${\mathscr{R}}\leq 1$ and some parameter limits are satisfied, the solution of model (3) oscillates around $E^{0}$. Significantly, the disease-free equilibrium $E^{0}$ of model (3) is stochastically asymptotically stable when $\sigma _{1}=0$ (see Theorem 3 and Remark 1). (iv) A sufficient condition is given for the existence of the stationary distribution by using the Khasminskii method. Under this sufficient condition, the solution of model (3) will oscillate around $E^{*}$ (see Theorem 4).

The numerical simulations are provided to illustrate the theoretical analysis. Take $\varphi (I)=1+I^{2}$. Four examples are given according to the following aspects: (i) Effects of the stochastic perturbations on the extinction of the infectious disease. (ii) Asymptotic behavior around the disease-free equilibrium. (iii) The existence of the stationary distribution. (iv) Effect of the partial immunization rate *p* on the disease-transmission dynamics. Through these numerical simulations, we observe that: (i) The sufficient condition for the extinction of the disease in model (3) is ${\mathscr{R}}^{s}<1$. Also, large perturbations may lead to the extinction of the disease even though it will be persistent in model (2). (ii) A large partial immunity rate can better control the epidemic than a small one.

In this work, we focus on using white noise to describe the randomness of the SIRS model. Other interesting topics for further work should be considered, such as a stochastic SIRS model with regime switching or Lévy Jumps. In recent years, the application of fractional differential equations in biological and epidemic models has increased significantly. Combined with the theory of fractional differential equations [46], the model can be extended to a fractional SIRS epidemic model. In addition, the partial-immune mechanism studied may also exist in some cells or viruses, which can be described by branching process [47]. We leave these for further investigation.

## Data Availability

Not applicable.

## Appendix

The proofs of the results (i) and (ii) of model (2) are provided as follows.

### Proof of (i)

The disease-free equilibrium $E^{0}(\frac{\Lambda}{\rho},0,0)$ can be directly obtained by setting $I=0$ in model (2). Next, we prove that if ${\mathscr{R}}< 1$, the disease-free equilibrium $E^{0}$ is globally asymptotically stable.

For convenience, we replace $(S,I,R)$ with $(N,I,R)$. Through model (2), this yields

$$ \mathrm{d}N=\mathrm{d}(S+I+R)=\Lambda -\rho N-\alpha I. $$

Obviously, model (2) is equivalent to the following model

20 $$ \textstyle\begin{cases} \frac{\mathrm{d}N}{\mathrm{d}t} =\Lambda -\rho N-\alpha I, \\ \frac{\mathrm{d}I}{\mathrm{d}t} =\frac{\beta I}{\varphi (I)}(N-I-R)-( \rho +\eta +\alpha )I, \\ \frac{\mathrm{d}R}{\mathrm{d}t} =p\eta I-(\rho +\theta )R. \end{cases} $$

Thus, the global stability of equilibria can be discussed by the model (20). Construct a function as

$$ V_{1}=\frac{\beta}{2\alpha} \biggl(N-\frac{\Lambda}{\rho} \biggr)^{2}+ \int _{0}^{I}\varphi (s)\,\mathrm{d}s+ \frac{\beta}{2p\eta}R^{2}. $$

Clearly, $V_{1}$ is a positive-definite function. The first-order derivative $\dot{V_{1}}$ of $V_{1}$ with respect to *t* is

$$\begin{aligned} \dot{V_{1}}&= \frac{\partial V_{1}}{\partial N} \frac{\mathrm{d}N}{\mathrm{d}t}+ \frac{\partial V_{1}}{\partial I} \frac{\mathrm{d}I}{\mathrm{d}t}+\frac{\partial V_{1}}{\partial R} \frac{\mathrm{d}R}{\mathrm{d}t} \\ &= \frac{\beta}{\alpha} \biggl(N-\frac{\Lambda}{\rho} \biggr) \biggl[- \rho \biggl(N-\frac{\Lambda}{\rho} \biggr)-\alpha I \biggr]+\varphi (I) \biggl[ \frac{\beta I}{\varphi (I)}(N-R-I)-(\rho +\eta +\alpha )I \biggr] \\ &\quad{}+\frac{\beta}{p\eta}R \bigl[p\eta I-(\rho +\theta )R \bigr] \\ &= -\frac{\beta \rho}{\alpha} \biggl(N-\frac{\Lambda}{\rho} \biggr)^{2}- \frac{\beta (\rho +\theta )}{p\eta}R^{2}-\beta I^{2}-(\rho +\eta + \alpha )I\varphi (I)+\frac{\Lambda}{\rho}\beta I \\ &= -\frac{\beta \rho}{\alpha} \biggl(N-\frac{\Lambda}{\rho} \biggr)^{2}- \frac{\beta (\rho +\theta )}{p\eta}R^{2}-\beta I^{2}-(\rho +\eta + \alpha )I\bigl(\varphi (I)-{\mathscr{R}}\bigr). \end{aligned}$$

The fact that $\varphi (0)=1$ and $f^{\prime}(I)\geq 0$ implies $\varphi (I)\geq 1$. Under ${\mathscr{R}}\leq 1$, one can easily obtain that $\dot{V_{1}}$ is negative-definite. By the Lyapunov asymptotic theorem, $E^{0}$ is globally asymptotically stable. □

### Proof of (ii)

To obtain the endemic equilibrium $E^{*}$, let the right sides of (2) be equal to zero, i.e.,

$$ \textstyle\begin{cases} \Lambda -\rho S^{*}-\frac{\beta S^{*}I^{*}}{\varphi (I^{*})}+(1-p) \eta I^{*}+\theta R^{*}=0, \\ \frac{\beta S^{*}I^{*}}{\varphi (I^{*})}-(\rho +\eta +\alpha )I^{*}=0, \\ p\eta I^{*}-(\rho +\theta )R^{*}=0. \end{cases} $$

Since $\varphi (0)=1$ and $f^{\prime}(I)\geq 0$, these equations can be simplified as

$$ \textstyle\begin{cases} S^{*}=\frac{\rho +\eta +\alpha}{\beta}\varphi (I^{*}), \\ R^{*}=\frac{p\eta}{\rho +\theta}I^{*}, \\ \Lambda -\rho S^{*}-(\rho +\eta +\alpha )I^{*}+(1-p)\eta I^{*}+ \theta R^{*}=0. \end{cases} $$

Substitute the first two equations into the third equation, i.e.,

$$ \Lambda -\frac{\rho (\rho +\eta +\alpha )}{\beta}\varphi \bigl(I^{*}\bigr)-( \rho +\eta +\alpha )I^{*}+(1-p)\eta I^{*}+ \frac{\theta p\eta}{\rho +\theta}I^{*}=0.$$

Define

$$\begin{aligned} T(I)=\Lambda -\frac{\rho (\rho +\eta +\alpha )}{\beta}\varphi (I)-\biggl( \rho +\alpha + \frac{p\eta \rho}{\rho +\theta}\biggr)I. \end{aligned}$$

Thus, $T(I^{*})=0$, that is, $I^{*}$ is the solution of the equation $T(I)=0$. Differentiating $T(I)$ with respect to *I*, we have

$$ T^{\prime}(I)=-\frac{\rho (\rho +\eta +\alpha )}{\beta}\varphi ^{ \prime}(I)-\biggl( \rho +\alpha +\frac{p\eta \rho}{\rho +\theta}\biggr). $$

Note that $\varphi '(I)\geq 0$, we have $T'(I)<0$. Thus, $T^{\prime}(I)$ decreases for $I\in [I(0),\infty )$. Based on the existence theorem of zero, Moreover, $T(I^{*})\leq \Lambda -(\rho +\alpha +\frac{p\eta \rho}{\rho +\theta})I^{*}$, which leads to $\lim_{I^{*}\rightarrow \infty}T(I^{*})=-\infty $. In addition,

$$ T(0)=\Lambda -\frac{\rho (\rho +\eta +\alpha )}{\beta}= \frac{\rho (\rho +\eta +\alpha )}{\beta}({\mathscr{R}}-1). $$

According to the existence theorem of the zero point, $T(I^{*})=0$ has a unique positive solution if and only if $T(0)>0$, i.e., ${\mathscr{R}}>1$. In other words, a unique endemic equilibrium $E^{*}(S^{*}, I^{*}, R^{*})$ exists in model (2).

Let $n=N-N^{*}$, $i=I-I^{*}$, and $r=R-R^{*}$. Substituting these variables into model (20), one can obtain the following equations

21 $$ \textstyle\begin{cases} \frac{\mathrm{d}n}{\mathrm{d}t} =-\rho n-\alpha i, \\ \frac{\mathrm{d}i}{\mathrm{d}t} = \frac{\beta (i+I^{*})}{\varphi (i+I^{*})} [n-i-r+S^{*} (1- \frac{\varphi (i+I^{*})}{\varphi (I^{*})} ) ], \\ \frac{\mathrm{d}r}{\mathrm{d}t} =p\eta i-(\rho +\theta )r. \end{cases} $$

Next, we only need to prove that the trivial solution of model (21) is globally asymptotically stable. Define

$$ \begin{aligned} V_{2}&= \frac{\beta}{2\alpha}n^{2}+ \varphi \bigl(I^{*}\bigr) \biggl[i-I^{*}\ln \biggl(1+ \frac{i}{I^{*}} \biggr) \biggr]+ \int _{I^{*}}^{i+I^{*}} \frac{(\varphi (s)-\varphi (I^{*}))(s-I^{*})}{s}\,\mathrm{d}s \\ &\quad{}+\frac{\beta}{2p\eta}r^{2}. \end{aligned} $$

Obviously, $V_{2}$ is a positive-definite function and

$$ \begin{aligned} \dot{V_{2}}&= \frac{\partial V_{2}}{\partial n} \frac{\mathrm{d}n}{\mathrm{d}t}+\frac{\partial V_{2}}{\partial i} \frac{\mathrm{d}i}{\mathrm{d}t}+ \frac{\partial V_{2}}{\partial r} \frac{\mathrm{d}r}{\mathrm{d}t} \\ &= \frac{\beta}{\alpha }n(-\rho n-\alpha i)+ \frac{\varphi (i+I^{*})}{i+I^{*}}i \biggl[ \frac{\beta (i+I^{*})}{\varphi (i+I^{*})} \biggl(n-i-r+S^{*} \biggl(1- \frac{\varphi (i+I^{*})}{\varphi (I^{*})} \biggr) \biggr) \biggr] \\ &\quad{}+\frac{\beta}{p\eta}r\bigl[p\eta i-(\rho +\theta )r\bigr] \\ &= -\frac{\beta \rho}{\alpha}n^{2}-\beta i^{2}- \frac{\beta (\rho +\theta )}{p\eta}r^{2}- \frac{\beta i}{\varphi (I^{*})}S^{*} \bigl(\varphi \bigl(i+I^{*}\bigr)-\varphi \bigl(I^{*} \bigr)\bigr). \end{aligned} $$

Recall that $f^{\prime}\geq 0$, one has $\varphi (i+I^{*})-\varphi (I^{*})\geq 0$. Evidently, $\dot{V_{2}}$ is negative-definite. Thus, $V_{2}$ is a Lyapunov function for model (21). By the Lyapunov asymptotic stability theorem, the trivial solution of model (21) is globally asymptotically stable. That is to say, the endemic equilibrium state $E^{*}(S^{*}, I^{*}, R^{*})$ is globally asymptotically. □
